# The Age-Accompanied and Diet-Associated Remodeling of the Phospholipid, Amino Acid, and SCFA Metabolism of Healthy Centenarians from a Chinese Longevous Region: A Window into Exceptional Longevity

**DOI:** 10.3390/nu14204420

**Published:** 2022-10-21

**Authors:** Da Cai, Zimo Zhao, Lingjun Zhao, Yanjie Dong, Lei Wang, Shancang Zhao, Quanyang Li

**Affiliations:** 1 Shandong Provincial Key Laboratory of Test Technology on Food Quality and Safety, Institute of Quality Standard and Testing Technology for Agro-Products, Shandong Academy of Agricultural Sciences, Jinan 250100, China; 2 College of Veterinary and Agriculture Science, University of Melbourne, Melbourne, VIC 3000, Australia; 3 Jinan Agro-Technical Extension and Service Center, Jinan 250004, China; 4 College of Light Industry and Food Engineering, Guangxi University, Nanning 530004, China

**Keywords:** centenarian, metabolomics, remodeling, phospholipid, amino acid, SCFA, dietary fiber, longevous region

## Abstract

As centenarians provide a paradigm of healthy aging, investigating the comprehensive metabolic profiles of healthy centenarians is of utmost importance for the pursuit of health and longevity. However, relevant reports, especially studies considering the dietary influence on metabolism, are still limited, mostly lacking the guidance of a model of healthy aging. Therefore, exploring the signatures of the integrative metabolic profiles of the healthy centenarians from a famous longevous region, Bama County, China, should be an effective way. The global metabolome in urine and the short-chain fatty acids (SCFAs) in the feces of 30 healthy centenarians and 31 elderly people aged 60–70 from the longevous region were analyzed by non-targeted metabolomics combined with metabolic target analysis. The results showed that the characteristic metabolites related to longevity were mostly summarized into phosphatidylserine, lyso-phosphatidylethanolamine, phosphatidylcholine, phosphatidylinositol, bile acids, and amino acids (*p* < 0.05). Six metabolic pathways were found significant relevant to longevity. Furthermore, acetic acid, propionic acid, butyric acid, valeric acid, and total SCFA were significantly increased in the centenarian group (*p* < 0.05) and were also positively associated with the dietary fiber intake (*p* < 0.01). It was age-accompanied and diet-associated remodeling of phospholipid, amino acid, and SCFA metabolism that expressed the unique metabolic signatures related to exceptional longevity. This metabolic remodeling is suggestive of cognitive benefits, better antioxidant capacity, the attenuation of local inflammation, and health-span-promoting processes, which play a critical and positive role in shaping healthy aging.

## 1. Introduction

Aging is a heterogeneous and complex process as many transformations happen to human organisms, such as a general decline in physiological function, increasing chronic low-grade inflammatory status, and increased risks of aging-related diseases. However, as an accepted model of successful aging, centenarians avoid or delay major age-related diseases, such as diabetes mellitus, Alzheimer disease, cardiovascular disease, and cancer [1]. Therefore, understanding the mechanism of exceptional longevity is of important referential significance for allowing populations to live longer, disease-free, and have a good quality of life. Decades of study on aging have found many genes and biological processes associated with the aging process [2], but its exact biological mechanism is still unclear. In particular, a general molecular profile that encompasses the healthy aging process as a result of multifactorial interactions is limited, and the data for centenarians are especially lacking.

Today, metabolomic approaches have become extremely promising tools for capturing overall metabolic changes associated with the normal aging process. Jové et al. found monoacylglyceride (22:1), diacylglyceride (33:2), resolvin D6, and phosphoserine (40:5) decreasing with the aging process by analyzing the metabolic profiles of healthy humans ranging from 30 to 100 years of age [3]. Chak et al. identified significant ageing-associated metabolites that are involved in several ageing processes, such as oxidative stress resistance, autophagy, inflammation, lipid metabolism, and apoptosis [4]. Bunning et al. applied random forest models to explore biological processes of aging in a cross-sectional cohort of healthy individuals aged 6 months to 82 years, which highlighted established metabolites, such as amino acids, steroids, and free fatty acids [5]. The above reports focused primarily on the normal aging process itself rather than the extreme longevity. The relevant reports on a comprehensive metabolic phenotype of centenarians are still relatively limited. Collino and Montoliu et al. performed metabolic profiling of Italian centenarians using NMR metabonomics and targeted analysis approaches and found that the centenarians possessed a unique eicosanoid metabolism network and identified phospho- and sphingolipids as markers of human longevity [1,6]. Nevertheless, as an important factor affecting metabolic profiles, the dietary influence has not yet been considered in both the researches. Furthermore, the exploration of integrative metabolic pathways closely related to extreme longevity in humans is still in its infancy so far, which, however, could provide insights into underlying molecular mechanisms and biological processes of healthy aging.

There is a remarkable phenomenon in Bama County, Guangxi Province, China. Based on the Population Census of China in 2020, there were 102 centenarians in the population of 236,152 in Bama County, a ratio of 43 centenarians per 1 × 10^5^ persons [7]. To date, the ratio of centenarians in this region is the highest in China, and is far above the world longevity county standard defined by the United Nations (7.5/100,000) [8]. The rare and amazing phenomenon of extreme longevity and healthy aging possesses distinctive local features, and therefore, the centenarians from this longevous region provide a valuable paradigm of healthy and successful aging for people to follow and imitate. Accordingly, we have reported the characteristics of nutrient intakes, specific metabolites and elements in nails of elderly people from the longevous region compared with a non-longevous region in previous studies [9,10]. However, no work has focused on the comprehensive metabolic profiles of centenarians living in Bama longevous region as a result of their traditional and conservative lifestyle.

Since our centenarians well represent a model of healthy and successful aging, the primary objective of this study is to discover the metabolic pattern of healthy aging by portraying the integrative metabolic profiles and then further capturing the metabolic signatures of the extreme longevity using the non-targeted metabolomics approach combined with metabolic target analysis. To achieve the aim, the healthy centenarians in the longevous region (LRC group) and the elderly people aged 60–70 in the longevous region (LRE group) were enrolled according to strict screening criteria. The characteristic metabolites and relevant metabolic pathways were identified based on the orthogonal projections to latent structures discriminant analysis (OPLS-DA) model. Moreover, the SCFA metabolism was analyzed. This will be very important for a better understanding and pursuit of longevity from the perspective of comprehensive metabolic profiles.

## 2. Materials and Methods

### 2.1. Participants

The study was carried out in Bama County, Guangxi Province, China. By means of thorough community screenings according to the population census data, we enrolled healthy centenarians in the longevous region (LRC group) and elderly people aged 60–70 in the longevous region (LRE group). The age of volunteers was validated according to our previous report [10]. To guarantee that each participant was relatively healthy, the screening criteria including rigorous ineligibility criteria were adopted based on the previous study [10]. During recruitment and screening, each volunteer was required to complete a questionnaire on medical history to provide health information.

All study procedures were reviewed and approved by the Ethics Committee of Guangxi University (approval no.: GXU-M-2019003). The study was conducted following the guidelines of the Declaration of Helsinki. Written informed consent was obtained from each participant.

### 2.2. Dietary Assessment

The dietary assessment was carried out by 4-season consecutive 7-day weighed dietary records (28-day WDRs) as described previously [10], which were performed in January, April, July, and October. According to the Chinese food composition tables, average daily intakes of energy, macronutrients, and dietary fiber were calculated by multiplying the quantities of food consumed (in g) or portion size by the contents of the above nutrients per 100 g of food listed in the Chinese food composition tables [11].

### 2.3. Sample Collection and Preparation

Fresh morning urine samples and fecal samples were collected and maintained at 4 °C for ≤5 h before processing, and then stored at −80 °C until analysis.

### 2.4. Non-Targeted Metabolomics Analysis Based on UPLC-MS

A 100 µL aliquot of each urine sample was thawed at room temperature, mixed with 400 µL ice-cold methanol, and vortexed for 30 s. Following centrifugation at 12,000 rpm for 15 min at 4 °C, the supernatant was filtrated by nylon syringe filters (0.22 μm pore size). Subsequently, 200 µL supernatant was transferred to autosampler vials and UPLC-MS analysis was performed.

The metabolomics analysis was conducted on an Ultimate 3000 LC coupled to an Orbitrap Elite mass spectrometry (MS) system (Thermo). A Hypergod C18 column (4.6 × 100 mm, 3 µm) was used. The column oven was set at 40 °C. The autosampler temperature was maintained at 4 °C. The gradient elution was performed using a mixture of solvent A (0.1% formic acid in water) and solvent B (0.1% formic acid in acetonitrile) at a flow rate of 0.3 mL/min. The starting conditions were 95% A and 5% B (*v*/*v*) for 2 min, shifting to 5% A and 95% B at 12 min and then keeping constant for 3 min. Subsequently, the solvent composition returned to the starting conditions at 17 min. A four-microliter sample was injected. To eliminate the effect of sample order, the injected samples were alternated between the LRC group and LRE group, and the sample sequence was random.

MS was performed in both the positive and negative ionization modes using an electrospray ionization source interface. The spray voltages were 3.0 kV in the ESI^+^ mode and 3.2 kV in the ESI^−^ mode. The capillary temperature was 350 °C, and the heater temperature was 300 °C. The sheath gas flow rate, aux gas flow rate, and sweep gas flow rate were 45 arbitrary units, 15 arbitrary units, and 1 arbitrary units, respectively. The mass scan range was set from 50 to 1000 *m*/*z*.

To ensure data quality of metabolomics analysis, quality control (QC) samples were used for column conditioning and method validation [12]. A QC sample was prepared by mixing equal amounts of each urine sample and processed using the same method used for sample preparation. Before analyzing the sample sequence, the QC sample was run three times. During the analysis of the sample sequence, 1 QC sample was run after every 10 injections. The repeatability and stability of the metabolomics analysis were determined by principal component analysis (PCA) of the whole dataset including all of the QC samples and were examined using 20 ions from the extracted ion chromatogram (XIC) of the QC samples.

### 2.5. Analysis of SCFAs in Feces

SCFAs including acetic acid, propionic acid, isobutyric acid, butyric acid, isovaleric acid, and valeric acid in feces were analyzed with capillary GC (GC-2010 Plus, Shimadzu, Kyoto, Japan) as described previously [10].

### 2.6. Data Processing and Statistical Analysis

The raw MS data were aligned using the SIEVE software package (Thermo) based on the *m*/*z* value and the retention time (RT). All of the detected ions in each sample were normalized to the sum of the peak areas. The data matrix including the sample name, the RT-*m*/*z* pair, the molecular weight, and the ion intensity was used for multivariate statistical analysis.

The data were imported into the SIMCA-P 16 program (Umetrics) for multivariate analysis. Data were scaled and logarithmically transformed to minimize the impacts of both noise and high variance of the variables. After these transformations, supervised OPLS-DA was applied. A sevenfold (Leave-1/7th Samples-Out) cross-validation procedure was carried out to avoid the risk of over-fitting. The parameters of the OPLS-DA model, such as the R^2^X, R^2^Y, and Q^2^Y, were analyzed to ensure the quality of the multivariate model.

The value of variable importance in the projection (VIP) in OPLS-DA analysis was used to identify the differential metabolites. The VIP statistics ranked the overall contribution of each variable to the OPLS-DA model, and those metabolites with VIP > 1.0 and *p* < 0.05 in Student’s *t*-test were considered as the significant differential metabolites. Furthermore, combined with the fold change (FC), the characteristic metabolites closely related to centenarians were determined. The heat map of characteristic metabolites was plotted using the R software package. Moreover, the Pearson correlation coefficients between metabolites were calculated and correlation network was constructed using the R software package.

The metabolic pathway analysis was performed using the KEGG database and MetaboAnalyst 5.0 (http://www.metaboanalyst.ca, accessed on 21 April 2022). As MetaboAnalyst assigns metabolites to their pathways using a limited database, not all of the metabolites can be analyzed to designate their pathways. However, MetaboAnalyst can provide valuable information of metabolic pathways by integrating two pathway analysis approaches, including pathway topology analysis and pathway enrichment analysis. Additionally, the results were visualized intuitively via a Google Maps-style visualization system.

Furthermore, a Spearman correlation test was used to evaluate correlations between dietary fiber intake and SCFAs in feces. The statistical significance was set at *p* < 0.01.

## 3. Results

### 3.1. Characteristics of the Participants

For this study, 49 centenarians and 56 elderly people from the longevous region were enrolled in the LRC group and LRE group, respectively. Of these, 37 centenarians in the LRC group and 45 elderly people in the LRE group met the screening criteria and then started this study. Among these volunteers, 30 centenarians in the LRC group and 31 elderly people in the LRE group completed the study protocol. As shown in Table 1, there is no significant difference in sex ratio between the two groups (*p* > 0.05).

### 3.2. Validation of Stability and Repeatability of Metabolomics Analysis

**Figure 1:**
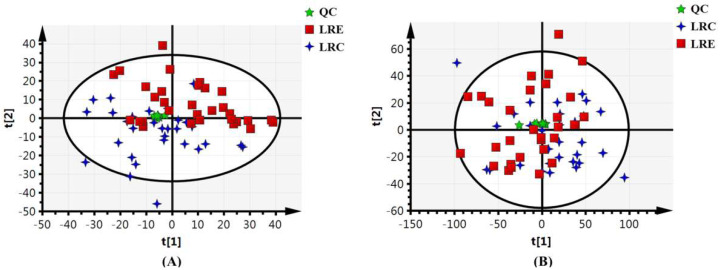
Scores plot from the principal component analysis of the overall data set containing all of the QC samples (9 replicates) (**A**) in the positive ionization mode; (**B**) in the negative ionization mode. Blue crosses represent LRC group. Red squares represent LRE group. Green asterisks represent QC samples. Their spatial distribution reveals the variations of the metabolic profiles. The closer the QC samples cluster on the score plot, the more stable the metabolomics analysis. All of the replicates of the QC samples within the analytical run are tightly clustered in the center, indicating that the metabolomics analysis in this study is stable and the differences between the samples are meaningful. QC, quality control; LRC, centenarians in the longevous region; LRE, elderly people aged 60–70 years in the longevous region.

In the metabolomics analysis based on UPLC-MS, QC samples were used to validate the stability and repeatability of the metabolomics analysis. As shown in Figure 1, all of the QC samples were tightly clustered in the center on the score plot from the principal component analysis of the dataset containing all of the QC samples. Since the closer QC samples cluster on a score plot, the more stable the analysis is [13], it was concluded that the analysis in this study was stable and the differences between the samples were meaningful. The ion intensity from the XIC of QC samples was examined (Table S1). The results showed that no significant variation was detected during the analysis process. The repeatability of ion intensity was satisfactory, with coefficients of variance (CV) in the range of 1.73–6.53%. Consequently, it was confirmed that the data of this metabolomics analysis were suitable to discover the metabolic pattern of healthy aging.

### 3.3. Global Metabolic Profiling of Urine

**Figure 2:**
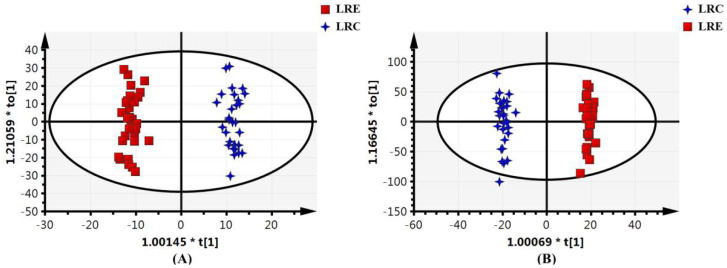
Scores plot from the orthogonal projections to latent structures discriminant analysis of the global metabolome in the LRC and LRE group (**A**) in the positive ionization mode; (**B**) in the negative ionization mode. Blue crosses represent LRC group. Red squares represent LRE group. Their spatial distribution reveals the variations of the metabolic profiles between the two groups. The two groups exhibit a clear separation, indicating that there are distinct differences in the global metabolic profiles between the two groups. LRC, centenarians in the longevous region; LRE, elderly people aged 60–70 years in the longevous region.

UPLC-MS data showed that a total of 2114 features and 7080 features were obtained in positive mode and negative mode, respectively. To further obtain a direct overview of the differences in global metabolic profiles between the two groups and discover the characteristic metabolites of healthy aging, the supervised OPLS-DA model was applied to the classification of the LRC group and LRE group, as the predictive components in OPLS-DA model can describe the effect of the healthy aging excluding the variance among samples in the same group. The OPLS-DA scores plots depicted a clear separation between the LRC group and LRE group (Figure 2). This suggests that there are distinct differences in the global metabolic profiles between the two groups.

The quality of the OPLS-DA models was examined using the R^2^Y and Q^2^Y values to verify that the models were not over-fitted and to evaluate the predictive ability of the models. R^2^Y represents the goodness-of-fit parameter, and Q^2^Y represents the predictive ability parameter. The OPLS-DA models with R^2^Y and Q^2^Y values greater than 0.5 are reliable mathematical models with satisfactory predictability. As shown in Table 2, in the ESI^+^ mode, R^2^Y and Q^2^Y were 0.984 and 0.801 respectively. In the ESI^−^ mode, R^2^Y and Q^2^Y were 0.993 and 0.796 respectively. Therefore, the results reveal that the OPLS-DA models were well-fitted and displayed a satisfactory predictive ability.

### 3.4. Identification of Characteristic Metabolites of the Centenarians

To extract potential variables contributing to the detected differences in global metabolic profiles between the two groups, the two criteria VIP > 1.0 in the OPLS-DA models and *p* < 0.05 in the *t*-test were applied to identify the differential metabolites. A total of 69 differential metabolites were obtained. The numbers of up-regulated and down-regulated metabolites in LRC group were 45 and 24, respectively. The differential metabolites were mostly classified into amino acids, organic acids, carbohydrates, bile acids, phosphatidylcholine (PC), phosphatidylserine (PS), lyso-phosphatidylethanolamine (Lyso PE), and phosphatidylinositol (PI).

**Figure 3:**
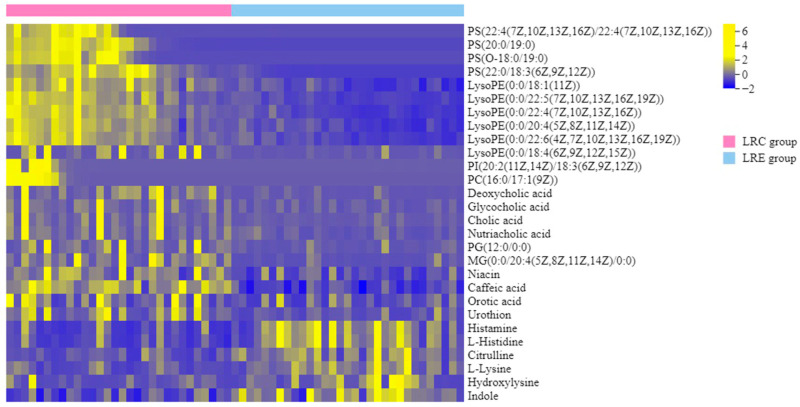
Heat map of the characteristic metabolites closely related to centenarians from the longevous region. The color scale correlates with the normalized quantities of the characteristic metabolites in samples: yellower color indicates higher quantities, and bluer color indicates lower quantities. Each row represents one characteristic metabolite, and each column represents one participant. Pink denotes the participants in the LRC group (left side), and blue denotes the participants in the LRE group (right side). It is more obvious to display a marked difference between the LRC group and LRE group, revealing some unique remodeling of amino acid metabolism and lipid metabolism of the healthy centenarians, compared with the elderly people aged 60–70 years. LRC, centenarians in the longevous region; LRE, elderly people aged 60–70 years in the longevous region.

To further investigate the characteristic metabolites closely related to centenarians from the longevous region, the fold change (FC) of metabolites in the LRC group versus the LRE group was used to screen the characteristic components. The variables with |log_2_FC| ≥ 1, VIP > 1.3 and *p* < 0.05 were designated as the characteristic metabolites. Consequently, 28 characteristic metabolites were obtained. The normalized quantities of the identified characteristic metabolites in the two groups were plotted in a heat map (Figure 3). The molecular weight, retention time, VIP in OPLS-DA models, *p* value in *t*-test, and FC of the characteristic metabolites are shown in Table 3. Among these metabolites, 6 metabolites including citrulline, lysine, hydroxylysine, histidine, histamine and indole were significantly decreased in the LRC group (*p* < 0.05), while 22 metabolites were significantly increased in the LRC group (*p* < 0.05). The up-regulated metabolites were mostly summarized into PS, Lyso PE, PC, PI, and bile acids. Of the 28 characteristic metabolites, the FC values of PS (O-18:0/19:0), PS (22:4/22:4) and PS (20:0/19:0) were highest, and were 12.892, 12.478, and 12.004, respectively. All of the three characteristic metabolites belong to PS. Additionally, the up-regulated characteristic metabolite Lyso PEs included Lyso PE (0:0/18:1), Lyso PE (0:0/22:5), Lyso PE (0:0/22:4), Lyso PE (0:0/20:4), Lyso PE (0:0/22:6), and Lyso PE (0:0/18:4). The up-regulated characteristic metabolite PC and PI were PC (16:0/17:1) and PI (20:2/18:3), respectively. The up-regulated characteristic metabolite bile acids included cholic acid, deoxycholic acid, glycocholic acid and nutriacholic acid. Among the down-regulated characteristic metabolites, the |FC| values of histamine, citrulline, hydroxylysine and L-histidine were highest, with FC values of −1.471, −1.449, −1.419, and −1.227, respectively. The heat map more obviously displayed a marked difference between the LRC group and LRE group. Overall, the results indicate that the specific metabolic profile of the centenarians from the longevous region reveals some unique and complex remodeling of amino acid metabolism and lipid metabolism, compared with the elderly people aged 60–70 years.

### 3.5. Correlation Relationships of Differential Metabolites in Urine

**Figure 4:**
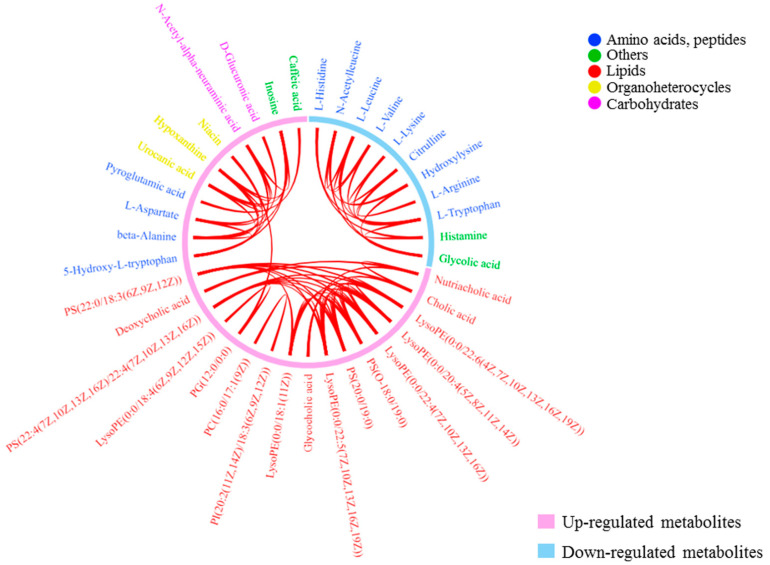
Chord diagram of the correlations between differential metabolites. When Pearson correlation coefficient between two metabolites is higher than 0.7 in absolute value, a connection is established. Colors denote the classification of metabolites. The pink circular arc represents up-regulated metabolites, and the blue circular arc represents down-regulated metabolites. More correlations between metabolites trend toward positive correlations. The most conspicuous correlations are lipid–lipid connections and amino acid-amino acid connections, revealing unique functional clusters of co-regulated metabolites including lyso-phosphatidylethanolamine, phosphatidylserine, bile acids, and amino acids.

To gain insight into how the above differential metabolites were coordinated in healthy aging, a chord diagram was constructed to investigate the latent relationships of the 69 differential metabolites. A connection was established between 2 metabolites when their Pearson correlation coefficient was higher than 0.7 in absolute value, as shown in Figure 4. The pink circular arc represented up-regulated metabolites, and the blue circular arc represented down-regulated metabolites. Interestingly, more correlations between metabolites trended toward positive correlations. The most conspicuous correlations were lipid-lipid connections and amino acid-amino acid connections, which revealed unique functional clusters of co-regulated metabolites including lyso-phosphatidylethanolamine, phosphatidylserine, bile acids, and amino acids. Among these correlations, the correlation coefficient of *N*-acetyl-α-neuraminic acid and inosine was highest (*R* = 0.9989, *p* = 3.7 × 10^−80^), followed by L-histidine and histamine (*R* = 0.9847, *p* = 1.7 × 10^−46^), nutriacholic acid and cholic acid (*R* = 0.9816, *p* = 3.8 × 10^−44^). Among the negative correlations, the absolute value of correlation coefficient of PG(15:1(9Z)/0:0) and 5-hydroxy-L-tryptophan was highest (*R* = −0.6977, *p* = 4.1 × 10^−10^).

### 3.6. Discovery of Metabolic Pathways Relevant to Healthy Aging

**Figure 5:**
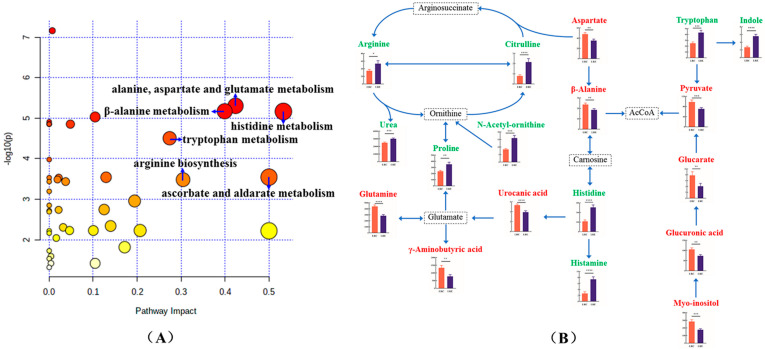
Metabolic pathways relevant to healthy aging. (**A**) Bubble diagram of pathway enrichment analysis (*y* axis) and pathway topology analysis (*x* axis) of differential metabolites. Circle size is proportional to pathway impact value, and darker red color indicates more significant differences. The names of the significantly relevant pathways in terms of impact values > 0.2 and adjusted *p*-values < 0.05 are highlighted, including alanine, aspartate and glutamate metabolism; β-alanine metabolism; histidine metabolism; tryptophan metabolism; ascorbate and aldarate metabolism; and arginine biosynthesis. (**B**) Schematic representation of integrative metabolic pathways, including the above 6 significantly relevant pathways. Significantly increased (red) and decreased (green) metabolites in the above significantly relevant pathways are shown. Column graphs show mean ± SEM of the differential metabolites in the LRC group (red bar) and LRE group (blue bar). * *p* < 0.05, ** *p* < 0.01, *** *p* < 0.001, **** *p* < 0.0001. Metabolites indicated in black represent that there is no significant difference between the two groups in this analysis. These significantly relevant metabolic pathways reflect a specific remodeling of amino acid metabolism in centenarians.

To identify the most relevant pathways of the above 69 differential metabolites, metabolic pathway analysis was performed using MetaboAnalyst 5.0, as well as the KEGG pathway database (http://www.genome.jp/kegg/, accessed on 26 April 2022), as shown in Figure 5A. The results of the pathway analysis are summarized in Table 4. By means of the pathway topology analysis, the results showed that the pathway impact values of eight metabolic pathways, including alanine, aspartate and glutamate metabolism; β-alanine metabolism; histidine metabolism; tryptophan metabolism; ascorbate and aldarate metabolism; arginine biosynthesis; pyruvate metabolism; and phenylalanine, tyrosine and tryptophan biosynthesis, were higher than 0.2, which was the cutoff value for relevance. Meanwhile, through the enrichment analysis, the results showed that alanine, aspartate and glutamate metabolism; β-alanine metabolism; histidine metabolism; tryptophan metabolism; ascorbate and aldarate metabolism; and arginine biosynthesis were significantly enriched, with adjusted *p*-values < 0.05. Thus, the above 6 metabolic pathways were considered to be the significantly relevant pathways in terms of impact values and adjusted *p*-values, which were closely related to centenarians from the longevous region. Among the significantly relevant metabolic pathways, the pathway impact value of histidine metabolism was highest (impact value 0.53), followed by ascorbate and aldarate metabolism (impact value 0.50); and alanine, aspartate and glutamate metabolism (impact value 0.42).

The schematic representation of integrative metabolic pathways including the above 6 significantly relevant pathways is shown in Figure 5B. Ascorbate and aldarate metabolism included myo-inositol, D-glucuronic acid and D-glucarate, in which all three metabolites significantly increased in the LRC group (*p* < 0.05). L-histidine, histamine, and urocanic acid belonged to histidine metabolism, in which L-histidine and histamine significantly decreased (*p* < 0.05) while urocanic acid significantly increased in the LRC group (*p* < 0.05), and urocanic acid was eventually converted to glutamate. In the pathway of alanine, aspartate and glutamate metabolism, L-glutamine, γ-aminobutyric acid, L-aspartate and pyruvate significantly increased in the LRC group (*p* < 0.05). L-aspartate, β-alanine and L-histidine were included in β-alanine metabolism, in which β-alanine also increased significantly in the LRC group (*p* < 0.05). Citrulline, L-arginine, *N*-acetyl-ornithine, and urea belonged to arginine biosynthesis, in which all of the four metabolites significantly decreased in the LRC group (*p* < 0.05). In the pathway of tryptophan metabolism, L-tryptophan and indole significantly decreased in the LRC group (*p* < 0.05). These significantly relevant metabolic pathways reflect a specific remodeling of amino acid metabolism in centenarians.

### 3.7. Diet-Associated Remodeling of SCFA Metabolism

**Figure 6:**
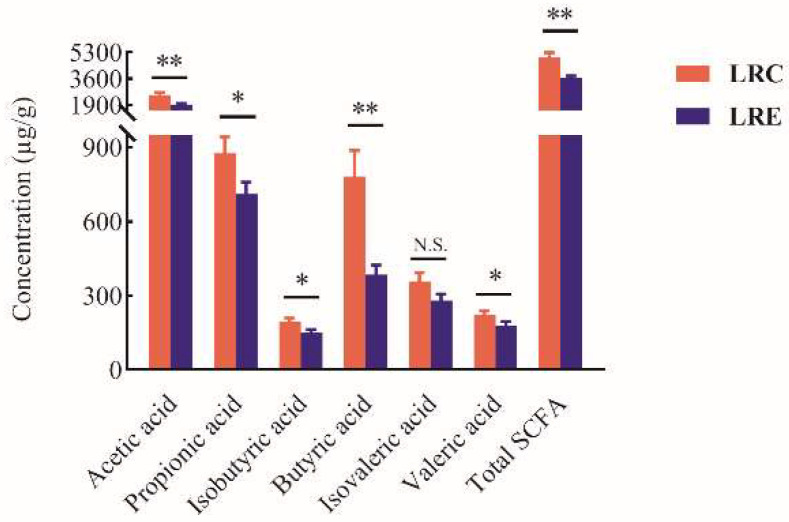
SCFAs in feces in the LRC group and LRE group. Data are shown as mean ± SEM. Asterisks designate significance by Student’s *t*-test. * *p* < 0.05, ** *p* < 0.01. N.S. denotes that there is no significant difference between the two groups. The concentrations of acetic acid, propionic acid, isobutyric acid, butyric acid, valeric acid, and total SCFA in the LRC group were significantly higher than those in the LRE group (*p* < 0.05), reflecting the unique remodeling of SCFA metabolism in the LRC group. LRC, centenarians in the longevous region; LRE, elderly people aged 60–70 years in the longevous region.

The contents of the SCFAs in feces are presented in Figure 6. The concentrations of acetic acid, propionic acid, isobutyric acid, butyric acid, valeric acid, and total SCFA in the LRC group were significantly higher than those in the LRE group (*p* < 0.05). The contents of acetic acid, propionic acid, isobutyric acid, butyric acid, valeric acid, and total SCFA increased 1.3-fold, 1.2-fold, 1.3-fold, 2.0-fold, 1.2-fold, and 1.4-fold, respectively, for the LRC group, compared with those for the LRE group. Consequently, the results revealed the unique remodeling of SCFA metabolism in the LRC group, indicating that higher contents of the SCFAs have a positive influence on health and longevity of the centenarians from the longevous region.

**Figure 7:**
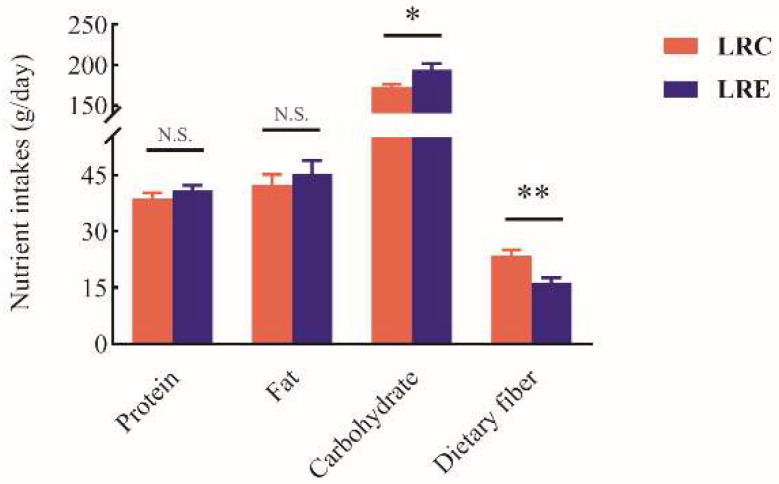
Macronutrient and dietary fiber intakes in the LRC group and LRE group. Data are shown as mean ± SEM. Asterisks designate significance by student *t*-test. * *p* < 0.05, ** *p* < 0.01. N.S. denotes that there is no significant difference between the two groups. The dietary fiber intake of the LRC group was significantly higher than that of the LRE group (*p* < 0.01). Meanwhile, the carbohydrate intake of the LRC group was significantly lower than that of the LRE group (*p* < 0.05). LRC, centenarians in the longevous region; LRE, elderly people aged 60–70 years in the longevous region.

In order to investigate why the above SCFAs were increased in the LRC group, the relationships between SCFAs and diet were further assessed. The intakes of macronutrients and dietary fiber in the LRC group and LRE group were obtained by 28-day WDR method coupled with the Chinese food composition tables, as shown in Figure 7. The dietary fiber intake of the LRC group was significantly higher than that of the LRE group (*p* < 0.01), which increased 1.4-fold for the LRC group compared with that for the LRE group. The carbohydrate intake of the LRC group was significantly lower than that of the LRE group (*p* < 0.05). There was no significant difference in protein and fat intake between the LRC group and LRE group. The energy intake and macronutrients-calorie percent composition were further calculated (Table 5). The results showed that energy intake of the LRC group was significantly lower than that of the LRE group (*p* < 0.01), whereas there were no significant differences in the energy supply ratios of protein, fat, and carbohydrate between the LRC group and LRE group. Therefore, this suggests that decreased energy intake and increased dietary fiber intake may be conducive to health and longevity.

Thus, the Spearman correlation test was performed to analyze the relationships between dietary fiber intake and SCFAs in feces, as shown in Table 6. Significant positive correlations were observed between the contents of acetic acid (*R* = 0.548, *p* < 0.01), propionic acid (*R* = 0.571, *p* < 0.01), butyric acid (*R* = 0.930, *p* < 0.01), valeric acid (*R* = 0.408, *p* < 0.01), and total SCFA (*R* = 0.724, *p* < 0.01) and the dietary fiber intake, which indicates that higher intake of dietary fiber makes for the elevated SCFAs in the feces of centenarians.

## 4. Discussion

Through the systematic analysis of comprehensive metabolic profiles of the healthy centenarians by the non-targeted metabolomics approaches coupled with the metabolic target analysis, we captured the unique metabolic signatures of the exceptional longevity, which achieved initial aim of this study and expanded our previous investigation on longevity characterization [10]. As centenarians provide an excellent paradigm of healthy aging, the exploration of metabolic patterns of healthy centenarians from the longevous region opens a window into extreme longevity. The relevant studies have elucidated some important metabolic alterations related to the aging process [1,3,4,5,6,14,15]. However, most of the reports lacked the guidance of a successful model of healthy aging. Only Collino and Montoliu et al. described the metabolic phenotype of Italian centenarians [1,6]. Despite these findings, the underlying metabolic pathways associated with these phenotypes as well as the dietary influence on the metabolism of centenarians have remained poorly understood. Nevertheless, we discovered the 28 characteristic metabolites and 6 metabolic pathways closely related to the centenarians for the first time, reflecting distinctive remodeling of phospholipid and amino acid metabolism. Moreover, we also demonstrated diet-associated remodeling of SCFA metabolism. These specific metabolic remodeling may play a critical role in shaping healthy aging.

It has been reported that phospholipids metabolism changed with aging, whereas there were some differences in individual phospholipid molecules among different studies [1,6,14,15,16], which could be attributed to the differences in geographic areas, genetic background, race, living environment, lifestyle, and dietary habits of the subjects. PC (14:0/18:1), PC (16:0/18:1), PC (16:0/18:2), PC (14:0/18:2), PC (16:0/18:3), PC (18:0/22:5), PE (16:0/20:4), PE (18:0/20:2), PE (18:0/20:3), PE (18:0/20:4), PI (18:0/18:1), PI (18:1/16:0), PI (20:3/18:0) were increased in Italian centenarians [6]. However, we found that the characteristic metabolites PS (O-18:0/19:0), PS (22:4/22:4), PS (20:0/19:0) and PS (22:0/18:3) were significantly increased in the LRC group (*p* < 0.05), especially PS (O-18:0/19:0), PS (22:4/22:4) and PS (20:0/19:0) with the highest FC. PSs have some potential cognitive benefits, which have been shown to increase memory performance in the elderly [17,18]. PSs are also widely involved in some important physiological processes, including phagocytosis by macrophage, and activation of protein kinase C [19]. This indicates that given the cognitive benefits, higher PSs should be beneficial to health and longevity, which also reflects the specific remodeling of phospholipids metabolism of the healthy centenarians from the longevous region.

The four bile acids, including cholic acid, deoxycholic acid, glycocholic acid, and nutriacholic acid, were significantly increased in the LRC group (*p* < 0.05), which were also characteristic metabolites closely related to the centenarians. Studies have demonstrated that bile acids act as metabolic regulators and nutrient sensors to regulate glucose and lipid metabolism, and immune response [20]. As signaling molecules, in mammals, bile acids specifically bind to and activate some receptors, hence stimulating many vital longevity-promoting and healthspan-promoting processes, such as anti-inflammatory processes [21]. Therefore, our finding suggests that the above four up-regulated bile acids may be beneficial to healthy aging. Notably, Zhang et al. found that the level of glycocholic acid was significantly decreased with age ranging from 20 to 74 years old [22]. In turn to see the discovery of this study, it is reasonably speculated that the bile acids metabolism of the healthy centenarians may revert to a more youthful state with respect to the elderly people aged 60–70 years, which also represents the unique metabolic signatures of the healthy centenarians from the longevous region, and a path to health and longevity by means of appropriately up-regulated bile acids metabolism.

The metabolic fate of the characteristic metabolite L-histidine has three possible routes. The first pathway is its conversion to the biogenic amine histamine. As a neurotransmitter, histamine is also involved in local immune responses [14]. The second pathway is its metabolism to urocanic acid. The third metabolic pathway that consumes L-histidine produces carnosine that is a dipeptide from β-alanine and L-histidine. Owing to its antioxidant characteristics, carnosine is considered to be a natural anti-aging substance capable of suppressing oxidative damage, glycation of proteins, and scavenging toxic age-related molecules [23]. From this point of view, the lower L-histidine levels in our study (*p* < 0.05) owing to its consumption by carnosine biosynthesis with advancing age might to some extent reflect a response to oxidative stress. A previous study showed that histidine decreased in serum with age (participants aged 32–81) [14]. However, we found that L-histidine and histamine significantly decreased in the urine of healthy centenarians (*p* < 0.05), displaying the unique and effective remodeling of histidine metabolism in centenarians to counteract oxidative stress.

We also discovered that the characteristic metabolite citrulline was significantly decreased in the LRC group (*p* < 0.05), which was involved in urea cycle that was down-regulated as well. In urea cycle, arginine level was also lower in the LRC group (*p* < 0.05). It has been shown that arginine was significantly decreased in ageing participants of KORA and CARLA [4]. Moreover, arginine level was recently shown to be significantly positively correlated with dietary carbohydrate intake (*R* = 0.79, *p* < 0.05) [24]. As for this study, we found that the carbohydrate intake was also significantly lower in the LRC group (*p* < 0.05), with a similar trend with that in the above report. Intriguingly, aspartate was significantly increased in the LRC group (*p* < 0.05). The relevant studies in cells and mammals have shown that aspartate supplementation reduces ROS production in neuroblastoma cells and reduces oxidative stress and increases antioxidant levels in the blood [25,26]. In fact, decreased ROS is believed to be a critical mechanism behind the extended lifespan and health span [27]. However, we have found no available data on relationships between aspartate and human longevity so far. Based on the discovery of this study, it is reasonable to assume that aspartate metabolism plays an important and positive role in human longevity.

As a nitrogen shuttle, glutamine takes up excess ammonia and forms urea, thereby reducing toxic build-up in the brain and improving brain functions [28]. Glutamine also plays an important role in NF-κB signal transduction pathways, contributing to the attenuation of local inflammation [6]. Montoliu et al. found that glutamine was increased in serum of Italian centenarians by ^1^H-NMR [6]. However, interestingly, we discovered a significant increase in glutamine in the urine of healthy centenarians from Bama County (*p* < 0.05). Given the physiological functions of glutamine metabolism, higher glutamine should have a positive influence on the health and longevity of centenarians.

In the pathway of ascorbate and aldarate metabolism, we observed a significant increased level of myo-inositol in the LRC group (*p* < 0.05). Myo-inositol promoted healthspan and prevented age-related decline in physiological functions in worm and mouse [29,30]. However, the relevant studies in humans have not been reported. Our finding suggests that increased myo-inositol may be conducive to longevity of centenarians.

As to tryptophan metabolism, a previous study showed that the tryptophan and indole concentrations in feces progressively decreased with age (volunteers aged 2 to 85 years) [31]. It has also been reported that tryptophan decreased in serum with increasing age [1]. Nevertheless, we found that tryptophan and its degradation product indole in urine of healthy centenarians were significantly down-regulated (*p* < 0.05) for the first time, indicating the characteristic remodeling of tryptophan metabolism of healthy centenarians from the longevous region. Additionally, their energy (*p* < 0.01) and carbohydrate (*p* < 0.05) intakes were significantly lower. Other work showed that tryptophan decreased with caloric restriction in an intervention study including eight subjects [32]. It was also reported that very low-carbohydrate ketogenic diet also decreased tryptophan levels in mice [33]. The above intervention study and mice experiment have similar trends with our results, suggesting that the specific remodeling of tryptophan metabolism may be associated with diet of centenarians.

We also discovered a particular remodeling of SCFA metabolism of healthy centenarians associated with dietary fiber intake (*p* < 0.01). It has been shown that oldest-old adults had greater potential for SCFA production [34]. Our previous study also found that SCFAs were increased in feces of the elderly people in Bama County, compared with the elderly people from a non-longevous region [10]. SCFAs perform various physiological functions in the gut, including anti-inflammatory, antimicrobial, and antitumorigenic effects [35], associated with lower risks for some diseases [36], especially butyric acid and propionic acid, promoting metabolic benefits via gut-brain neural circuits [37]. It is therefore concluded that the relatively higher levels of SCFAs may be conducive to longevity of the centenarians, and appropriate increased dietary fibers in daily diets should be a path toward the longevity.

This study has several strengths. First, the two complementary strategies, non-targeted metabolomics and metabolite target analysis were used. Non-targeted metabolomics is a valuable approach to obtaining a comprehensive depiction of the metabolic status closely related to the phenotypic outcome of interest in an unbiased manner [38]. Meanwhile, targeted metabolomics, which is an accurate quantitative method to analyze biochemically known and annotated metabolites, provides information that is more precise on specific metabolites and metabolic pathways [39]. Second, the two kinds of different biological samples, including urine and feces, were analyzed to comprehensively assess the metabolic features of healthy centenarians from different perspectives. To date, most of the relevant reports primarily focus on metabolites in serum and plasma [3,4,5,6,14,15,16,40]. Collino et al. explored the metabolic changes in serum and urine of Italian centenarians [1]. However, the investigation on the signatures of global metabolic profiles of the exceptional longevity using two kinds of biological samples—urine and feces—by means of the two complementary metabolomics approaches has not been reported yet so far. Third, the four-season consecutive 7-day WDR method was used to assess the usual habitual nutrient intakes, which reflects real-life nutrient intakes and minimizes variances in dietary intakes according to seasons and days. Among available nutrition assessment approaches, the WDR method is the most accurate and robust and is accepted as a gold standard [41], though it is expensive, time-consuming, and generally requires considerable commitment on the part of volunteers [42]. Fourth, characteristic metabolites and metabolic pathways closely related to centenarians from the longevous region were discovered based on the OPLS-DA model that is reliable and robust for a small sample size.

Nevertheless, some limitations of this exploratory study need to be noted. First, healthy aging also depends on many other factors, such as hereditary and environmental factors [43], while this study is limited by its cross-sectional design, and therefore a causal relationship between metabolism and longevity cannot be concluded directly. However, the unique metabolic signatures of the centenarians from the longevous region discovered in this study provided new clues for further exploration of the relationship between metabolism and longevity. Future studies are needed to investigate the metabolic mechanisms affecting healthy aging. Second, the detailed data on the smoking history and amount were unavailable, and therefore, it is necessary to exclude the influence of smoking in future work. Third, the strict screening criteria of participants, as well as an extremely limited number of healthy centenarians, resulted in a relatively small sample size. In addition, due to the tedious process of the 28-day WDR method and the complicated protocols of the sample collection, many volunteers withdrew from the study. Nevertheless, we applied the OPLS-DA method to construct the classification model of the LRC group and LRE group and identify characteristic metabolites. As a pattern recognition approach, OPLS-DA has distinct advantages in solving the classification problem of a very small number of samples [44], which overcomes the limitation of sample size. Further studies including additional cohorts from different longevous regions are required to validate these findings.

## 5. Conclusions

The unique age-accompanied and diet-associated remodeling of phospholipid, amino acid, and SCFA metabolism in healthy centenarians from the longevous region opens a window into the extreme longevity. Given the specific physiological functions of the characteristic metabolites and the relevant metabolic pathways, this metabolic remodeling is suggestive of the cognitive benefits, better antioxidant capacity, attenuation of local inflammation, and health-span-promoting processes, which plays a critical and positive role in shaping healthy aging. These findings help to pave a new avenue for further understanding human longevity from a metabolic point of view.

## Figures and Tables

**Table 1:** Characteristics of the participants.

Characteristics	LRC Group	LRE Group
Age (year)	103 ± 3	63 ± 3
Sex (male/female)	11/19	12/19
Height (cm)	145.9 ± 10.6	153.7 ± 7.2
Weight (kg)	43.1 ± 10.0	49.4 ± 9.4
Body mass index (kg/m^2^)	20.0 ± 2.8	20.8 ± 2.9

Values are means ± standard deviation (SD). LRC, centenarians in the longevous region; LRE, elderly people aged 60–70 years in the longevous region.

**Table 2:** The parameters of the OPLS-DA model.

	Component	R^2^X (cum)	R^2^Y (cum)	Q^2^Y (cum)
ESI^+^ mode	4P+1O	0.329	0.984	0.801
ESI^−^ mode	5P+1O	0.434	0.993	0.796

R^2^X (cum) and R^2^Y (cum) represent the cumulative modeled variation in X and Y matrices, respectively, and Q^2^Y (cum) is the cumulative predicted variation. 4P+1O, four predictive components and one orthogonal component for establishing the OPLS-DA model in the ESI^+^ mode. 5P+1O, five predictive components and one orthogonal component for establishing the OPLS-DA model in the ESI^−^ mode.

**Table 3:** Characteristic metabolites of the centenarians from the longevous region.

Metabolites	Molecular Weight	Retention Time	VIP	*p*	FC	Change Trend
PS(22:4(7Z,10Z,13Z,16Z)/22:4(7Z,10Z,13Z,16Z))	887.5598	11.82	2.361	<0.001	12.478	↑
PS(20:0/19:0)	833.6138	12.50	2.922	<0.001	12.004	↑
PS(O-18:0/19:0)	791.6030	7.77	2.641	<0.001	12.892	↑
PS(22:0/18:3(6Z,9Z,12Z))	841.5833	8.48	3.275	<0.001	4.824	↑
LysoPE(0:0/18:1(11Z))	479.3020	8.02	3.044	<0.001	1.154	↑
LysoPE(0:0/22:5(7Z,10Z,13Z,16Z,19Z))	527.3031	7.83	3.062	<0.001	2.630	↑
LysoPE(0:0/22:4(7Z,10Z,13Z,16Z))	529.3183	8.12	3.255	<0.001	2.189	↑
LysoPE(0:0/20:4(5Z,8Z,11Z,14Z))	501.2858	7.31	3.005	<0.001	1.066	↑
LysoPE(0:0/22:6(4Z,7Z,10Z,13Z,16Z,19Z))	525.2857	7.27	2.949	<0.001	1.948	↑
LysoPE(0:0/18:4(6Z,9Z,12Z,15Z))	473.2537	4.31	1.588	0.004	3.118	↑
PI(20:2(11Z,14Z)/18:3(6Z,9Z,12Z))	884.5386	8.26	1.416	0.010	10.920	↑
PC(16:0/17:1(9Z))	745.5593	9.96	1.612	0.003	9.817	↑
Deoxycholic acid	392.2937	5.76	1.673	0.002	3.204	↑
Glycocholic acid	465.3101	4.33	1.424	0.010	1.709	↑
Cholic acid	408.2888	4.74	1.447	0.009	2.910	↑
Nutriacholic acid	390.2742	4.74	1.397	0.006	1.987	↑
PG(12:0/0:0)	428.2236	6.35	1.444	0.009	2.348	↑
MG(0:0/20:4(5Z,8Z,11Z,14Z)/0:0)	378.2778	6.25	1.908	<0.001	3.062	↑
Niacin	123.0325	1.24	2.013	<0.001	1.059	↑
Caffeic acid	180.0418	1.86	3.143	<0.001	1.134	↑
Orotic acid	156.0172	0.90	1.384	0.012	1.012	↑
Urothion	324.1676	3.45	1.655	0.001	1.770	↑
Histamine	111.0801	0.82	2.233	<0.001	−1.471	↓
L-Histidine	155.0702	0.77	2.340	<0.001	−1.227	↓
Citrulline	175.0965	0.86	1.869	0.001	−1.449	↓
L-Lysine	146.1060	0.86	1.691	0.002	−1.199	↓
Hydroxylysine	162.1011	0.75	1.348	0.015	−1.419	↓
Indole	117.0543	0.86	2.338	<0.001	−1.057	↓

Variable importance in the projection (VIP) is obtained from OPLS-DA with a threshold of 1.3. The *p* values are obtained by *t*-test. The fold change (FC) is calculated by logarithmic value of the ratio of the LRC group to LRE group. Change trend represents change trend of the metabolite in the LRC group as compared to the LRE group. The ↑ and ↓ represent that the metabolites are increased and decreased in the LRC group compared with the LRE group, respectively.

**Table 4:** Metabolic pathways of differential metabolites.

Pathway Name	Total	Hits	Raw *p*	Holm Adjusted *p*	Impact
Alanine, aspartate and glutamate metabolism	28	4	4.96 × 10^−6^	0.0002	0.42
β-Alanine metabolism	21	3	6.75 × 10^−6^	0.0003	0.40
Histidine metabolism	16	4	6.85 × 10^−6^	0.0003	0.53
Tryptophan metabolism	41	2	3.14 × 10^−5^	0.0010	0.27
Ascorbate and aldarate metabolism	8	3	0.0003	0.0089	0.50
Arginine biosynthesis	14	6	0.0003	0.0089	0.30
Pyruvate metabolism	22	1	0.0059	0.0828	0.21
Phenylalanine, tyrosine and tryptophan biosynthesis	4	1	0.0060	0.0828	0.50

Total denotes the total number of metabolites in the pathway. Hits denotes the actually matched number of metabolites in the pathway. Raw *p* is the original *p* value calculated from the enrichment analysis. Holm-adjusted *p* is the *p* value adjusted by Holm–Bonferroni method. Impact represents the pathway impact value calculated from pathway topology analysis.

**Table 5:** Energy intake and macronutrients-calorie percent composition.

	LRC Group	LRE Group	*p*
Energy (Kcal)	1220.30 ± 134.60 ^a^	1349.42 ± 97.67 ^b^	<0.001
Protein-calorie percent composition	12.72% ± 1.56% ^a^	12.11% ± 1.99% ^a^	0.188
Fat-calorie percent composition	30.72% ± 7.97% ^a^	29.91% ± 11.37% ^a^	0.748
Carbohydrate-calorie percent composition	56.90% ± 6.33% ^a^	57.89% ± 12.54% ^a^	0.699

Values are means ± SD. The *p* values are obtained by *t*-test. Values without a common superscript letter in a row are significantly different. LRC, centenarians in the longevous region; LRE, elderly people aged 60–70 years in the longevous region.

**Table 6:** Correlations between dietary fiber intake and short chain fatty acids in feces.

	Acetic Acid	Propionic Acid	Isobutyric Acid	Butyric Acid	Isovaleric Acid	Valeric Acid	Total SCFA
*R*	0.548 **	0.571 **	0.219	0.930 **	0.112	0.408 **	0.724 **
*p*	<0.001	<0.001	0.089	<0.001	0.392	0.001	<0.001

*R* denotes the Spearman correlation coefficient. ** represents *p* < 0.01, correlations are statistically significant at the 0.01 level.

## Data Availability

The data in this study are available on request from the corresponding author.

## Supplementary Materials

The following supporting information can be downloaded at: https://www.mdpi.com/article/10.3390/nu14204420/s1, Table S1: Repeatability of ion intensity in QC samples.
