# Supplementary material for: The Age-Accompanied and Diet-Associated Remodeling of the Phospholipid, Amino Acid, and SCFA Metabolism of Healthy Centenarians from a Chinese Longevous Region: A Window into Exceptional Longevity
Source: Nutrients. 2022 Oct 21;14(20):4420. doi: 10.3390/nu14204420 (PMC9612356; doi:10.3390/nu14204420)
Supplement: Supplementary file 1 [file nutrients-14-04420-s001.zip › nutrients-1930311-supplementary.pdf]

Table S1 Repeatability of ion intensity in QC samples

Molecular weight	Retention time	m/z	Ion intensity	CV
Positive mode				
117.05	0.86	118.06	160.42	2.55%
131.07	0.87	132.08	862.65	1.81%
103.06	0.89	104.07	864.93	3.01%
60.03	0.94	61.04	24523.75	5.64%
113.06	0.95	114.07	14164.04	3.60%
137.05	0.97	138.05	778.79	1.96%
129.04	1.22	130.05	443.87	3.12%
131.06	1.23	132.07	45.79	5.64%
182.04	1.47	183.05	170.50	5.02%
220.08	1.55	221.09	376.08	3.66%
180.04	1.86	181.05	151.31	4.54%
179.08	2.41	180.09	395.78	4.75%
204.09	3.35	188.07	1737.25	2.32%
180.05	3.48	181.06	490.16	4.90%
146.07	3.70	130.05	3975.81	1.85%
394.25	3.86	395.25	8.31	4.89%
594.34	4.12	595.35	122.31	3.43%
465.31	4.36	466.31	119.71	4.51%
267.09	4.53	268.10	44.02	3.26%
317.29	5.51	318.30	1484.55	2.11%
Negative mode				
155.07	0.77	154.06	186.77	3.19%
136.04	0.87	135.03	1338.40	2.45%
166.05	0.87	165.04	397.19	5.11%
194.04	0.87	193.04	121.45	2.90%
133.04	0.89	132.03	55.85	2.51%
180.06	0.89	179.06	312.16	3.40%
156.02	0.90	155.01	14.36	6.53%
150.05	0.97	149.05	416.18	3.02%
89.05	1.20	245.08	26.42	6.16%
123.03	1.24	122.03	11.65	5.78%
160.07	2.18	159.07	142.11	4.16%
158.06	3.45	157.05	196.99	1.74%
181.07	3.46	180.07	16.24	4.13%
132.08	3.88	172.10	78.73	5.62%
173.11	3.88	172.10	52.34	4.67%
594.34	4.11	593.34	5.87	6.30%
465.31	4.33	464.30	389.92	1.73%
481.24	4.35	480.23	74.74	3.66%
267.09	4.52	266.09	48.23	3.00%
408.29	4.74	453.29	259.63	2.22%

CV, coefficient of variance.
